# Sociodemographic, Lifestyle, and Social Isolation Correlates of TyG, METS-IR, and SPISE-IR Scores in a Large Spanish Working Population

**DOI:** 10.3390/medsci13030171

**Published:** 2025-09-03

**Authors:** Pere Riutord-Sbert, Pedro Juan Tárraga López, Ángel Arturo López-González, Irene Coll Campayo, Carla Busquets-Cortés, José Ignacio Ramírez Manent

**Affiliations:** 1 ADEMA-Health Group, University Institute for Research in Health Sciences (IUNICS), 07010 Palma, Spaini.coll@eua.edu.es (I.C.C.);; 2 Faculty of Medicine, UCLM (University of Castilla La Mancha), SESCAM (Health Service of Castilla La Mancha), 02008 Albacete, Spain; pjtarraga@sescam.jccm.es; 3 Balearic Islands Health Research Institute Foundation (IDISBA), 07010 Palma, Spain; 4 Balearic Islands Health Service, 07010 Palma, Spain; 5 Faculty of Medicine, University of the Balearic Islands, 07010 Palma, Spain

**Keywords:** insulin resistance, social isolation, TyG indez, Mediterranean diet, physical activity, sociodemographic variables

## Abstract

**Background:** Insulin resistance (IR) is a central feature in the pathophysiology of type 2 diabetes and a major determinant of cardiovascular morbidity. While sociodemographic and lifestyle factors are established contributors, the role of social isolation as a potential determinant of IR remains underexplored in working populations. **Objectives:** To assess the association between sociodemographic variables, lifestyle habits, and social isolation with three validated insulin resistance indexes—Triglyceride–Glucose (TyG), Metabolic Score for Insulin Resistance (METS-IR), and Single Point Insulin Sensitivity Estimator (SPISE-IR)—in a large cohort of Spanish workers. **Methods:** A cross-sectional study was conducted involving 117,298 workers from occupational health centers across Spain. Sociodemographic data, lifestyle factors (Mediterranean diet adherence, physical activity, and smoking), and social support levels (ENRICHD Social Support Instrument) were recorded. Biochemical and anthropometric parameters were obtained through standardized protocols. Logistic regression models estimated the odds ratios (ORs) and 95% confidence intervals (CIs) for high IR risk across the three indexes, adjusting for potential confounders. **Results:** Male sex, older age, lower social class, smoking, low Mediterranean diet adherence, physical inactivity, and low social support were independently associated with higher odds of IR in all three indexes. The strongest associations were observed for physical inactivity (OR range 6.21–9.95) and low social support (OR range 1.98–3.76). Although effect sizes varied by index, patterns of association were consistent. **Conclusions:** Sociodemographic, lifestyle, and psychosocial factors, including social isolation, are strongly associated with insulin resistance in working populations. Integrating social support assessment into occupational health strategies may enhance early detection and prevention of IR and related cardiometabolic diseases.

## 1. Introduction

Insulin resistance (IR) is a prevalent metabolic disturbance characterized by a diminished biological response of peripheral tissues—particularly skeletal muscle, liver, and adipose tissue—to circulating insulin [1]. IR underpins the pathophysiology of major chronic conditions, including type 2 diabetes mellitus, metabolic syndrome, non-alcoholic fatty liver disease, and cardiovascular disease [2,3,4,5], and is implicated in certain cancers and cognitive decline [6,7]. Mechanistically, IR arises from an interplay of genetic predisposition, adiposity, chronic inflammation, oxidative stress, endoplasmic reticulum stress, mitochondrial dysfunction, and lipotoxicity [8,9,10,11,12]. At a systemic level, IR impairs insulin-mediated glucose uptake, leading to compensatory hyperinsulinemia, dysglycemia, dyslipidemia, endothelial dysfunction, and pro-thrombotic states [13,14]. Epidemiologically, IR affects a substantial portion of adult populations worldwide—prevalence often exceeds 30%, with increases linked to obesity and aging trends [15].

Given the impracticality of using the hyperinsulinemic-euglycemic clamp in large-scale studies [16], surrogate indices such as HOMA-IR, QUICKI [17], triglyceride–glucose (TyG) index, METS-IR, and SPISE-IR are widely utilized [18,19,20]. These scores correlate well with gold-standard measures and allow risk stratification in epidemiological settings [21,22]. However, few studies have concurrently compared multiple IR indices—including TyG, METS-IR, and SPISE-IR—within the same cohort, particularly among working-age populations in Southern Europe.

In addition to classical metabolic and demographic risk factors (e.g., age, sex, BMI, and socioeconomic status), psychosocial determinants have garnered attention for their role in metabolic health. Notably, social isolation—the objective state of having minimal social contacts or engagement—has increasingly been recognized as a key determinant of both mental and physical health outcomes [23,24]. Distinct from perceived social support, social isolation refers to an actual deficiency of social interactions, often resulting in loneliness, decreased social participation, and reduced access to informal care [25]. Surveys indicate that 10–30% of working-age adults in Spain and other European countries experience social isolation, depending on measurement criteria [26], and in the elderly population, this prevalence is higher [27,28].

The health repercussions of social isolation are broad: it is associated with increased risks of cardiovascular disease, all-cause and specific mortality, depression, cognitive decline, and accelerated biological aging [29,30,31,32,33]. Mechanistically, social isolation may elevate stress and inflammation, promote unhealthy behaviors, and erode adherence to health-promoting routines [34,35,36]. In the metabolic domain, there is growing evidence linking social isolation and low social support to dysglycemia and adverse diabetes outcomes, mediated by behavioral and neuroendocrine pathways [37]. For example, low social support has been associated with elevated HbA_1_c in individuals with diabetes, partly due to reduced adherence to diet, physical activity, and medication regimens [38].

Valid measurement of social isolation and support requires reliable tools. The ENRICHD Social Support Instrument (ESSI) is one such brief self-report measure, developed in the U.S. as part of the ENRICHD trial. It comprises five items assessing emotional and instrumental support, one item on marital/partner status, and one item on social network size [39]. ESSI has demonstrated solid psychometric properties and predictive validity across diverse groups, such as older adults [40], and has begun to be adapted for European populations, including Spain—though evidence in working-age populations remains limited.

Against this backdrop, our study aims to examine the associations between sociodemographic factors, health-related behaviors (especially Mediterranean diet adherence and physical activity), and social isolation (measured via ESSI) with three IR risk indices—TyG, METS-IR, and SPISE-IR—in a large cohort of Spanish workers. This multidimensional approach addresses existing research gaps, as most IR studies focus predominantly on traditional metabolic drivers and frequently overlook the psychosocial context.

In Spain’s occupational health environment, large workplace cohorts are increasingly utilized to monitor chronic disease risk. Incorporating psychosocial instruments such as ESSI into these settings is both innovative and pertinent. While prior studies have investigated IR in relation to sociodemographic and lifestyle variables, the simultaneous evaluation of multiple IR indices alongside validated measures of social isolation in working-age adults remains scarce. Bridging this gap could pave the way for comprehensive, psychosocially informed, workplace-based prevention strategies.

## 2. Methods

### 2.1. Study Design and Population

**Figure 1:**
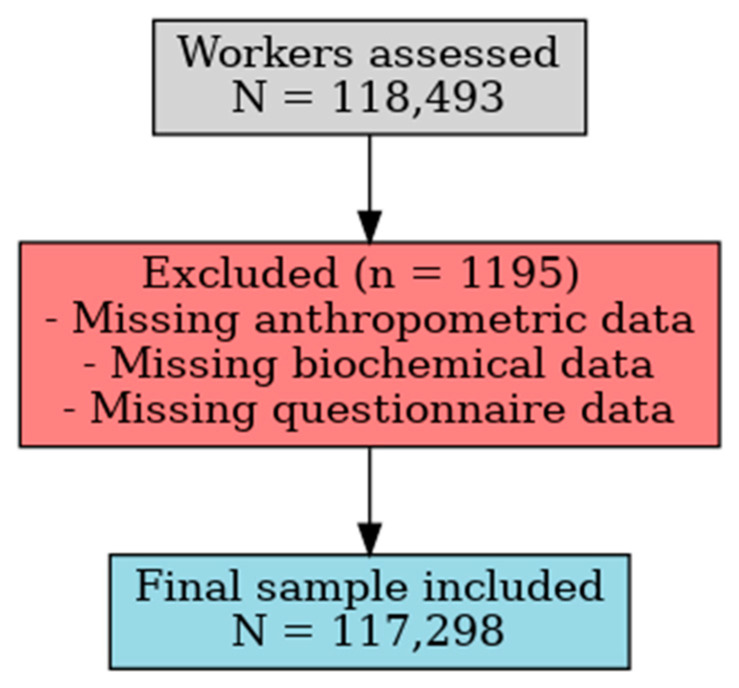
Flowchart of Participant Selection.

This cross-sectional study was conducted within an occupational health surveillance framework in Spain between January 2021 and December 2024. A total of 118,493 workers underwent routine medical evaluations as part of their annual occupational health assessments. After applying inclusion and exclusion criteria, 1195 participants were excluded due to incomplete clinical, biochemical, or questionnaire data. The final sample comprised 117,298 individuals (both sexes, aged 18–69 years) included in the analysis (Figure 1).

### 2.2. Inclusion and Exclusion Criteria

Eligible participants were men and women aged 18–69 years who completed standardized occupational health check-ups during the study period. Exclusion criteria included missing data for anthropometric measurements, biochemical parameters, insulin resistance (IR) indices, or social isolation assessment. These criteria are consistent with previous large-scale occupational cohort studies in Spain [41].

### 2.3. Sociodemographic Variables

Demographic data included sex, age (categorized as 18–39, 40–49, 50–59, or 60–69 years), and social class. Social class was determined according to the Spanish National Classification of Occupations (CNO-11) and the criteria of the Spanish Society of Epidemiology, grouping participants into Class I (highest), II, and III (lowest) [42].

### 2.4. Lifestyle Habits

Lifestyle information was obtained through structured questionnaires. **Physical activity** was assessed using the short form of the **International Physical Activity Questionnaire (IPAQ)**, validated for the Spanish population, which evaluates activity in the last seven days across work, transport, household, and leisure time domains. Without differentiating between occupational and recreational domains, this may limit its ability to capture habitual patterns. Responses were converted into metabolic equivalent task minutes per week (MET-min/week) and categorized into low, moderate, or high activity levels according to IPAQ scoring protocols [43,44].

It is important to further specify how ‘physical activity’ should be interpreted in this occupational cohort. The IPAQ-SF questionnaire assesses the frequency and intensity of activity over the previous seven days, without distinguishing between occupational physical effort and leisure time or recreational activity. Substantial differences exist depending on the type of occupation: office employees, whose work involves administrative tasks and prolonged computer use, generally perceive physical activity as an additional effort outside of their working hours. Conversely, workers in physically demanding sectors, such as construction, manufacturing, or farming, integrate moderate-to=vigorous physical activity as part of their daily occupational routine. This occupational heterogeneity may influence both the reporting and perception of physical activity levels and should therefore be considered when interpreting the study results. We also acknowledge that this limitation may introduce misclassification bias, as the IPAQ-SF does not allow for differentiation between work-related and leisure time activity.

**Dietary adherence** was assessed using the 14-item Mediterranean Diet Adherence Screener (MEDAS-14), with scores <9 indicating low adherence [45,46]. **Smoking status** was classified as a current smoker or a non-smoker.

#### 2.4.1. Anthropometric and Biochemical Measures

Weight and height were measured with participants wearing light clothing and no shoes, using calibrated scales and stadiometers. Body mass index (BMI) was calculated as weight (kg)/height (m^2^). Waist circumference was measured at the midpoint between the lower rib margin and the iliac crest, using a non-elastic tape. After an overnight fast of at least 8 h, venous blood samples were drawn to determine plasma glucose, triglycerides, and HDL-cholesterol. All biochemical analyses were performed in accredited laboratories following standardized enzymatic and colorimetric methods.

#### 2.4.2. Insulin Resistance Indices

Three surrogate IR indices were calculated:

- **Triglyceride–glucose (TyG) index** = ln [fasting triglycerides (mg/dL) × fasting glucose (mg/dL)/2], originally described by Nayak et al. [47] and validated in different populations.
- **Metabolic Score for Insulin Resistance (METS-IR)** and **Single Point Insulin Sensitivity Estimator for IR (SPISE-IR)**, calculated according to published formulas, previously applied in Spanish occupational cohorts [48,49].
- METS-IR was calculated using the following formula: Metabolic score for insulin resistance (METS-IR). METS-IR = Ln (2 × glucose) + triglycerides × BMI)/(Ln (HDL-c). High values are defined as 50 and above.
- SPISE was obtained using the validated formula: Single-Point insulin Sensitivity estimator (SPISE-IR). SPISE (=600 × HDL0.185/triglycerides0.2 × BMI1.338).
- For interpretative consistency with TyG and METS-IR, we defined SPISE-IR as 10/SPISE, so that higher values indicate greater insulin resistance.
- SPISE-IR = 10/SPISE is considered high risk at 1.51.
- For harmonization with TyG and METS-IR, we defined SPISE-IR as 10/SPISE, so that higher values reflect greater insulin resistance.

#### 2.4.3. Social Isolation Assessment

Social isolation was assessed using the **ENRICHD Social Support Instrument (ESSI)**, which consists of five items measuring emotional and instrumental support, one item on marital status, and one on the size of the social network. Items are scored on a Likert scale, with lower scores indicating reduced perceived support. In this study, participants were classified into “normal” or “low” social isolation based on established cut-offs [50]. In our coding, ‘low social isolation’ refers to low ESSI scores, which reflect reduced perceived social support and greater social vulnerability

#### 2.4.4. Statistical Analysis

All statistical analyses were performed using **SPSS version 29.0** (IBM Corp., Armonk, NY, USA). Continuous variables were summarized as means ± standard deviations (SD), and categorical variables were summarized as frequencies and percentages. Between-group differences were assessed using Student’s *t*-test, ANOVA, or the chi-square test, as appropriate. Multivariate logistic regression models were used to estimate odds ratios (OR) and 95% confidence intervals (CIs) for high-risk IR categories (TyG, METS-IR, and SPISE-IR), adjusting for sociodemographic, lifestyle, and social isolation variables. Statistical significance was set at *p* < 0.05.

## 3. Results

Table 1 comprehensively summarizes the anthropometric, clinical, and lifestyle characteristics of the 117,298 participants, stratified by sex. Statistically significant differences (*p* < 0.001 for all variables) are evident in virtually all measured parameters, highlighting a clear sexual dimorphism in body composition, metabolic profile, and health behaviors. Men exhibited higher mean values for height, weight, waist circumference, blood pressure, LDL-c, triglycerides, and fasting glucose, whereas women presented higher HDL-c levels and a healthier lifestyle profile, including greater adherence to the Mediterranean diet and higher rates of physical activity. Notably, a markedly higher prevalence of low social isolation was observed in men compared to women (38.4% vs. 9.1%), suggesting potential psychosocial disparities that may influence metabolic outcomes.

Table 2 illustrates the graded associations between key demographic and lifestyle factors and the continuous values of the three insulin resistance risk scores. Across both sexes, older age groups, lower social class, smoking, non-adherence to the Mediterranean diet, physical inactivity, and low social isolation were consistently associated with higher TyG and METS-IR values and lower SPISE-IR values, all in directions indicative of greater insulin resistance. The magnitude of differences was particularly striking for lifestyle-related variables, such as Mediterranean diet adherence and physical activity, which showed the largest absolute differences in score values, underscoring their strong potential as modifiable determinants of insulin resistance.

Table 3 presents prevalence estimates for participants exceeding established thresholds for each index. The data reveal consistent and clinically relevant patterns: the prevalence of high-risk values increased with age, lower social class, smoking, non-adherence to the Mediterranean diet, and physical inactivity. Low social isolation showed one of the strongest associations, with prevalence rates up to 41.8% for high TyG in men and 32.4% in women. Conversely, adherence to healthy lifestyle behaviors, such as regular physical activity and a Mediterranean dietary pattern, was associated with substantially lower prevalence, reinforcing the protective nature of these behaviors against insulin resistance.

Table 4 quantifies the independent associations between each sociodemographic, lifestyle, and social isolation variable and the likelihood of exhibiting high-risk values for the three indices, adjusted for potential confounders. Physical inactivity and non-adherence to the Mediterranean diet emerged as the most powerful predictors across all indices, with odds ratios exceeding 6.0 for physical inactivity in TyG and METS-IR, and nearly 10.0 for SPISE-IR. Low social isolation was also a robust predictor, with odds ratios ranging from 1.98 to 3.76, indicating that psychosocial determinants are comparably relevant to traditional metabolic risk factors. The consistency of associations across all three indices strengthens the validity of these findings.

**Figure 2:**
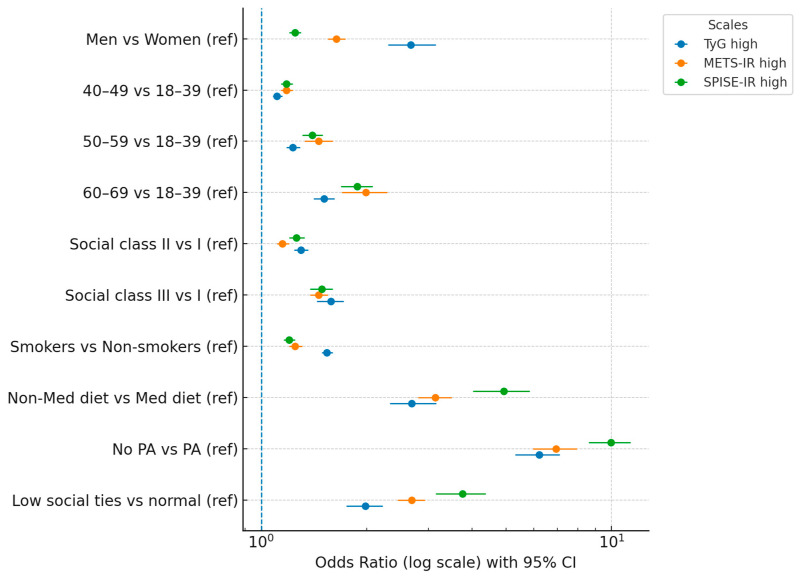
Forest plot of adjusted odds ratios for high TyG, METS-IR, and SPISE-IR values.

Figure 2 visually synthesizes the multivariable-adjusted odds ratios from Table 4, enabling rapid comparison of the magnitude and direction of associations across the three indices. The log-scale representation facilitates interpretation of wide-ranging effect sizes, and the parallel plotting of the three indices in different colors highlights similarities and discrepancies in their sensitivity to specific determinants. The figure clearly depicts the particularly large effects of physical inactivity, non-adherence to the Mediterranean diet, and low social isolation, which stand out as the dominant risk factors in the studied population.

**Figure 3:**
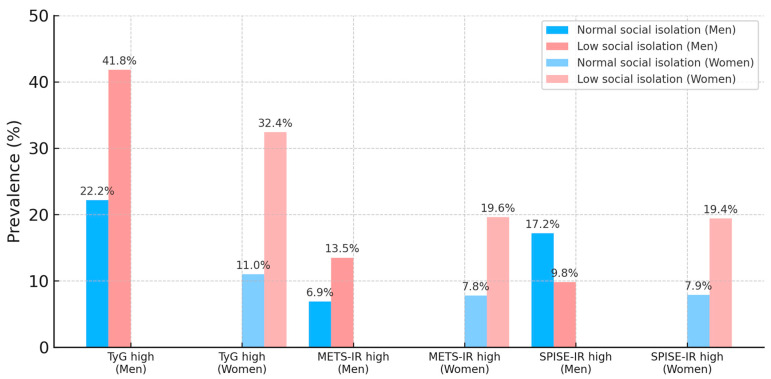
Prevalence of high TyG, METS-IR, and SPISE-IR values by social isolation status and sex.

Figure 3 illustrates the sex-stratified differences in prevalence according to social isolation status. In both men and women, low social isolation was associated with markedly higher prevalence of high TyG and METS-IR values, whereas for SPISE-IR, the pattern was more heterogeneous between sexes. The graphical representation emphasizes the interplay between psychosocial and biological determinants of insulin resistance risk, and the sex differences suggest that interventions aimed at reducing social isolation may need to be tailored differently for men and women to maximize their metabolic benefits.

## 4. Discussion

### 4.1. Principal Findings

In this very large occupational cohort of Spanish workers (*n* = 117,298), we observed consistent, dose-responsive associations between sociodemographic and lifestyle factors and the probability of presenting high values in three non-insulin insulin-resistance (IR) risk indices (TyG, METS-IR, and SPISE-IR). Male sex and older age showed graded increases in IR risk; lower social class, current smoking, low Mediterranean diet adherence, and physical inactivity were all independently associated with higher odds of TyG-high and METS-IR-high and with lower insulin sensitivity as captured by SPISE-IR. Among modifiable factors, physical inactivity and poor diet quality showed the largest effect sizes across indices. Importantly, low social isolation (worse social connectedness) was robustly and independently related to IR risk for all three indices, adding a salient psychosocial dimension to the IR profile in working-age adults.

### 4.2. Comparison with the Literature

Our results align with—and extend—prior evidence on the external validity and clinical relevance of TyG, METS-IR, and SPISE-IR. For TyG, several meta-analyses and large cohort studies show strong prospective associations with incident type 2 diabetes (T2D) and other adverse outcomes, supporting its utility for population risk-stratification. For example, a 2024 umbrella review/meta-analysis reported pooled relative risks >3 for T2D in higher TyG categories and also linked TyG to GDM and diabetic retinopathy, highlighting its broad prognostic scope [51,52]. Our findings that METS-IR tracks higher diabetes risk are consistent with contemporary cohort data showing that higher METS-IR predicts new-onset T2D and deteriorating glycemic status in the general population, beyond traditional confounders [53,54].

SPISE-IR—although less widely used in occupational epidemiology—showed patterns congruent with lower insulin sensitivity. Recent analyses indicate that SPISE performs well (and in some contexts better than alternatives) for identifying metabolic syndrome and dysglycemia in adults and youth [55,56,57], which supports its inclusion alongside TyG and METS-IR to triangulate IR risk. Moreover, beyond glycemia, higher METS-IR has been associated with cardiometabolic prognosis (e.g., mortality) [58], emphasizing that these indices capture risk dimensions relevant to downstream events.

Regarding lifestyle, our data showing the strongest detrimental associations for physical inactivity are concordant with recent meta-analyses of randomized trials in sedentary adults: combined aerobic-plus-resistance training significantly improves HOMA-IR, inflammatory cytokines, and HbA_1_c versus controls [59,60]. Complementary meta-analytic evidence shows that exercise training (aerobic and combined modalities) lowers fasting glucose, insulin, and HOMA-IR, with additional support for resistance-training–specific benefits in adults with overweight/obesity [61,62]. Our observation that low adherence to a Mediterranean-type diet relates to higher IR risk mirrors the clinical and observational literature wherein Mediterranean-diet exposure associates with lower IR and improved cardiometabolic biomarkers [63,64]. Smoking’s positive association with IR is also aligned with epidemiologic data linking chronic smoking exposure to increased odds of insulin resistance after multivariable adjustment [65].

Notably, we document independent links between social isolation and IR across three indices. While few studies have directly connected objective isolation with validated non-insulin IR scores, our findings are consistent with large prospective cohorts showing that social isolation and loneliness predict incident T2D, even after extensive adjustment [66,67], and with studies relating isolation/loneliness to major adverse cardiovascular events [68]. Although the term ‘low social isolation’ could semantically suggest better social integration, in this study, it denotes reduced social support and thus greater vulnerability. This distinction is essential to interpret why this category was associated with a worse metabolic profile.

These observations suggest plausible behavioral, neuroendocrine, and inflammatory pathways that converge on insulin resistance. Mechanistically, population-scale proteomic work identified signatures of isolation/loneliness enriched in inflammatory and antiviral pathways and linked prospectively to CVD, T2D, stroke, and mortality [69]. Finally, the educational gradients observed herein (higher risk in lower social class) parallel recent European evidence demonstrating pronounced educational inequalities in metabolic syndrome across the life course [70,71].

It is important to note that all participants in our cohort had a body mass index (BMI) greater than 27 kg/m^2^, indicating overweight or obesity. In this context, insulin resistance should be interpreted primarily as a consequence of excess adiposity rather than its cause. This clarification is essential for an accurate interpretation of the associations observed between insulin resistance indices and sociodemographic, lifestyle, and psychosocial variables.

### 4.3. Contributions of This Study

This investigation contributes in four main ways. First, it simultaneously benchmarks three complementary IR indices (TyG, METS-IR, and SPISE-IR) within the same very large, working-age cohort, enabling cross-index comparison of effect sizes across identical covariates—something infrequently reported. Second, it integrates a validated psychosocial construct (social isolation, via ESSI) into occupational IR epidemiology, showing independent, consistent associations even after adjusting for sociodemographic and lifestyle determinants. Third, it emphasizes modifiable levers—physical activity and diet adherence—as the strongest correlates across indices, aligning epidemiology with intervention targets demonstrated to be effective in trials [72]. Fourth, by documenting coherent patterns across TyG, METS-IR, and SPISE-IR, it supports the pragmatic use of multi-index screening to reduce misclassification and to capture complementary facets of IR biology relevant for prevention and surveillance.

### 4.4. Perspectives and Implications

Our results support workplace-embedded, multicomponent prevention that jointly addresses physical inactivity, dietary quality, and social connectedness. Feasible strategies include structured exercise programs during work hours, on-site or app-assisted Mediterranean diet counseling, and initiatives to foster social ties, which may yield synergistic benefits on IR biology. Given emerging evidence that METS-IR predicts incident diabetes and that social isolation relates to T2D incidence and adverse cardiovascular endpoints, occupational health systems could incorporate periodic calculation of IR indices plus brief social isolation screening to identify high-risk workers early and tailor interventions. Mechanistic advances from proteomic studies should inform future trials that test whether improving social connection alongside exercise/diet modifies inflammatory proteomic signatures and downstream IR trajectories.

### 4.5. Strengths and Limitations

Strengths include the following:

One of the main strengths of this study is the use of a robust and standardized methodology, which includes validated protocols for clinical and biochemical data collection, as well as internationally recognized instruments (IPAQ-SF for physical activity, MEDAS-14 for adherence to the Mediterranean diet, and ESSI for social isolation). This methodological approach provides consistency and reliability to the findings

Other strengths of this study are as follows:

(a) the very large sample, enhancing precision and enabling robust sex-stratified and age-stratified analyses; (b) the standardized clinical/laboratory protocols used in occupational health surveillance; (c) triangulation across three IR indices, which reduces reliance on any single surrogate; (d) comprehensive adjustment for sociodemographic and lifestyle covariates; and (e) use of a validated instrument for social isolation (ESSI).

Limitations merit careful consideration.

The cross-sectional design precludes causal inference and temporal ordering; longitudinal follow-up is needed to establish whether social isolation and lifestyle factors precede changes in IR indices and incident diabetes.

Residual confounding is possible; observational analyses cannot rule out all confounders.

Self-report for lifestyle and social isolation may introduce misclassification. The use of the IPAQ-SF represents a methodological limitation, as it reflects only the previous seven days and may be influenced by temporary circumstances, failing to capture long-term habitual activity. Moreover, it does not differentiate between occupational and leisure time activity, which is particularly relevant in a heterogeneous working population including both office employees and manual laborers. This limitation may lead to misclassification bias and should be considered when interpreting the results.

Generalizability: The findings may not extend to unemployed or clinically managed populations

Laboratory variability could introduce non-differential errors.

Index definitions and cut-offs: Although TyG, METS-IR, and SPISE-IR are validated surrogates, thresholds may vary by population.

Potential selection bias if workers with worse health were less likely to attend screening.

Lack of direct insulin measures limits physiologic granularity; nonetheless, accumulating evidence supports the prognostic utility of these surrogates.

Although the ESSI is a validated and useful instrument to assess perceived social support, its limitations should be acknowledged. The scale focuses on emotional and instrumental support, marital status, and network size but does not capture key dimensions such as relationship quality, community participation, or digital interactions. Moreover, it does not differentiate between in-person and virtual support, which may be particularly relevant among young adults and in contexts where socialization occurs predominantly online.

The binary categorization of social isolation into ‘normal’ and ‘low’ represents a simplification of a complex construct. While this approach facilitates epidemiological analysis, it may fail to capture intermediate gradations that are relevant for understanding the phenomenon. Future studies should consider more detailed or multidimensional scales to better reflect the diversity of social contexts

## 5. Conclusions

In this large occupational cohort of 117,298 Spanish workers, higher values of TyG, METS-IR, and SPISE-IR scores were significantly associated with male sex, older age, lower social class, smoking, poor adherence to the Mediterranean diet, physical inactivity, and low social support as assessed by means of the ENRICHD Social Support Instrument (ESSI). These associations were consistent across the three insulin resistance indexes, although the magnitude of effect varied, with physical inactivity and low social support showing the strongest relationships.

Our findings reinforce the relevance of integrating sociodemographic and lifestyle factors, as well as psychosocial determinants such as social isolation, into preventive strategies aimed at reducing insulin resistance and related cardiometabolic disorders in working populations. The inclusion of social support assessments in occupational health programs could help identify vulnerable groups and optimize interventions.

Future longitudinal studies are needed to confirm causal relationships and explore the underlying mechanisms linking social isolation with insulin resistance. Such evidence would strengthen the case for comprehensive, multidimensional prevention strategies targeting both behavioral and psychosocial risk factors.

## Figures and Tables

**Table 1:** Baseline characteristics of the study population by sex.

	Men *n* = 71,384	Women *n* = 45,914	
Variables	Mean (SD)	Mean (SD)	*p*-Value
Age (years)	45.5 (7.4)	45.2 (7.2)	<0.001
Height (cm)	173.1 (7.0)	160.2 (6.5)	<0.001
Weight (kg)	82.2 (13.5)	66.0 (12.9)	<0.001
Waist (cm)	88.5 (9.2)	74.4 (7.9)	<0.001
Hip (cm)	100.5 (8.3)	97.7 (8.7)	<0.001
Systolic BP (mm Hg)	126.4 (15.7)	116.7 (15.4)	<0.001
Diastolic BP (mm Hg)	77.4 (10.6)	71.3 (10.5)	<0.001
Cholesterol (mg/dL)	205.0 (37.3)	201.4 (36.0)	<0.001
HDL-c (mg/dL)	49.5 (6.9)	52.6 (7.4)	<0.001
LDL-c (mg/dL)	129.1 (36.6)	130.7 (36.4)	<0.001
Triglycerides (mg/dL)	133.4 (92.1)	91.1 (48.4)	<0.001
Glucose (mg/dL)	90.0 (13.2)	85.8 (11.8)	<0.001
Variables	*n* (%)	*n* (%)	*p*-value
18–39 years	18,418 (25.8)	12,214 (26.6)	<0.001
40–49 years	32,098 (45.0)	20,934 (45.6)	
50–59 years	17,350 (24.5)	11,094 (24.2)	
60–69 years	3338 (4.7)	1672 (3.6)	
Social class I	4002 (5.6)	2980 (6.5)	<0.001
Social class II	12,978 (18.2)	13,856 (30.2)	
Social class III	54,404 (76.2)	29,078 (63.3)	
Smokers	24,426 (34.2)	14,132 (30.8)	<0.001
Yes Mediterranean diet	22,858 (32.0)	20,536 (44.7)	<0.001
Yes physical activity	26,010 (36.4)	20,478 (45.2)	<0.001
Social isolation low	27,376 (38.4)	4198 (9.1)	<0.001
Social isolation normal	44,008 (61.6)	41,716 (90.9)	

BP, Blood pressure. HDL, High-density lipoprotein. LDL, Low-density lipoprotein. SD, Standard deviation.

**Table 2:** Mean values of TyG, METS-IR, and SPISE-IR by sociodemographic, lifestyle, and social isolation categories.

		TyG		METS-IR		SPISE-IR	
	N	Mean (SD)	*p*-Value	Mean (SD)	*p*-Value	Mean (SD)	*p*-Value
18–39 years	18,418	8.4 (0.6)	<0.001	38.7 (7.3)	<0.001	1.7 (0.5)	<0.001
40–49 years	32,098	8.5 (0.6)		40.3 (7.4)		1.8 (0.5)	
50–59 years	17,350	8.6 (0.6)		42.0 (7.2)		1.9 (0.5)	
60–69 years	3338	8.6 (0.5)		42.7 (6.6)		1.9 (0.4)	
Social class I	4002	8.4 (0.5)	<0.001	39.4 (6.8)	<0.001	1.7 (0.4)	<0.001
Social class II	12,978	8.5 (0.6)		39.9 (7.3)		1.8 (0.5)	
Social class III	54,404	8.6 (0.6)		40.6 (7.5)		1.8 (0.5)	
Smokers	24,426	8.6 (0.6)	<0.001	40.6 (7.1)	<0.001	1.8 (0.5)	<0.001
Non-smokers	46,778	8.4 (0.6)		40.1 (7.9)		1.7 (0.5)	
Yes Mediterranean diet	22,858	8.2 (0.4)		34.5 (3.4)		1.4 (0.2)	
Non Mediterranean diet	48,346	8.7 (0.6)		43.2 (7.1)		2.0 (0.5)	
Yes physical activity	26,010	8.2 (0.4)	<0.001	34.4 (3.4)	<0.001	1.4 (0.2)	<0.001
Non physical activity	45,194	8.7 (0.6)		43.8 (6.9)		2.0 (0.4)	
Social isolation low	27,376	8.7 (0.6)	<0.001	46.1 (6.8)	<0.001	2.1 (0.5)	<0.001
Social isolation normal	44,008	8.4 (0.5)		36.9 (5.3)		1.6 (0.3)	
	N	Mean (SD)	*p*-Value	**Mean (SD)**	***p*-Value**	**Mean (SD)**	***p*-Value**
18–39 years	12,214	8.0 (0.5)	<0.001	34.4 (7.8)	<0.001	1.4 (0.5)	<0.001
40–49 years	20,934	8.1 (0.5)		36.0 (7.7)		1.5 (0.5)	
50–59 years	11,094	8.3 (0.5)		38.4 (7.6)		1.6 (0.5)	
60–69 years	1672	8.4 (0.5)		39.4 (7.6)		1.7 (0.5)	
Social class I	2980	8.0 (0.4)	<0.001	33.6 (6.8)	<0.001	1.3 (0.4)	<0.001
Social class II	13,856	8.1 (0.5)		34.5 (7.2)		1.4 (0.4)	
Social class III	29,078	8.2 (0.5)		37.4 (8.0)		1.6 (0.5)	
Smokers	14,132	8.2 (0.5)	<0.001	36.7 (7.9)	<0.001	1.6 (0.5)	<0.001
Non-smokers	31,781	8.1 (0.5)		35.4 (7.5)		1.5 (0.5)	
Yes Mediterranean diet	20,536	7.9 (0.4)		31.3 (3.6)		1.2 (0.2)	
Non Mediterranean diet	25,377	8.3 (0.5)		40.3 (8.0)		1.8 (0.5)	
Yes physical activity	20,478	7.9 (0.4)	<0.001	30.9 (3.4)	<0.001	1.2 (0.2)	<0.001
Non physical activity	25,155	8.4 (0.5)		40.7 (7.7)		1.8 (0.5)	
Social isolation low	4198	8.5 (0.5)	<0.001	47.0 (7.7)	<0.001	2.2 (0.5)	<0.001
Social isolation normal	41,716	8.1 (0.5)		35.2 (7.0)		1.5 (0.4)	

TyG, Triglyceride glucose index. METS-IR, Metabolic score for insulin resistance. SPISE-IR, Single point for insulin sensitivity–insulin resistance. SPISE-IR = 10/SPISE. SD, Standard deviation.

**Table 3:** Prevalence of high TyG, METS-IR, and SPISE-IR values according to sociodemographic, lifestyle, and social isolation categories.

		TyG High		METS-IR High		SPISE-IR High	
	*n*	%	*p*-Value	%	*p*-Value	%	*p*-Value
18–39 years	18,418	22.7	<0.001	7.5	<0.001	12.7	<0.001
40–49 years	32,098	30.4		9.9		16.9	
50–59 years	17,350	34.7		12.7		19.5	
60–69 years	3338	35.2		13.0		19.6	
Social class I	4002	22.4	<0.001	8.3	<0.001	12.3	<0.001
Social class II	12,978	28.3		8.6		17.1	
Social class III	54,404	30.6		10.6		17.4	
Smokers	24,426	34.1	<0.001	11.2	<0.001	17.7	<0.001
Non-smokers	46,778	27.5		9.6		16.0	
Yes Mediterranean diet	22,858	10.3		5.1		7.9	
Non Mediterranean diet	48,346	30.6		16.9		17.5	
Yes physical activity	26,010	9.0	<0.001	4.0	<0.001	6.8	<0.001
Non physical activity	45,194	34.8		17.8		20.6	
Social isolation low	27,376	41.8	<0.001	13.5	<0.001	9.8	<0.001
Social isolation normal	44,008	22.2		6.9		17.2	
	*n*	%	*p*-Value	%	*p*-Value	%	*p*-Value
18–39 years	12,214	7.4	<0.001	4.7	<0.001	6.6	<0.001
40–49 years	20,934	11.6		6.1		8.4	
50–59 years	11,094	20.0		7.4		11.1	
60–69 years	1672	24.9		9.7		15.1	
Social class I	2980	7.7	<0.001	3.2	<0.001	4.5	<0.001
Social class II	13,856	10.2		4.4		6.0	
Social class III	29,078	14.8		7.3		10.6	
Smokers	14,132	14.8	<0.001	6.7	<0.001	9.6	<0.001
Non-smokers	31,781	12.1		5.0		7.2	
Yes Mediterranean diet	20,536	7.1		3.5		5.5	
Non Mediterranean diet	25,377	15.2		7.9		11.0	
Yes physical activity	20,478	6.0	<0.001	3.0	<0.001	4.0	<0.001
Non physical activity	25,155	18.2		8.6		13.5	
Social isolation low	4198	32.4	<0.001	19.6	<0.001	19.4	<0.001
Social isolation normal	41,716	11.0		7.8		7.9	

TyG, Triglyceride glucose index. METS-IR, Metabolic score for insulin resistance. SPISE-IR, Single point for insulin sensitivity–insulin resistance. SPISE-IR = 10/SPISE.

**Table 4:** Adjusted odds ratios for high TyG, METS-IR, and SPISE-IR values.

	TyG High		METS-IR High		SPISE-IR High	
	OR (95% CIs)	*p*-Value	OR (95% CIs)	*p*-Value	OR (95% CIs)	*p*-Value
Women	1		1		1	
Men	2.67 (2.30–3.15)	<0.001	1.64 (1.55–1.74)	<0.001	1.25 (1.20–1.30)	<0.001
18–39 years	1		1		1	
40–49 years	1.11 (1.08–1.15)	<0.001	1.18 (1.14–1.23)	<0.001	1.18 (1.14–1.23)	<0.001
50–59 years	1.23 (1.18–1.29)	<0.001	1.46 (1.33–1.60)	<0.001	1.40 (1.31–1.50)	<0.001
60–69 years	1.51 (1.41–1.62)	<0.001	1.99 (1.70–2.29)	<0.001	1.88 (1.69–2.08)	<0.001
Social class I	1		1		1	
Social class II	1.30 (1.24–1.36)	<0.001	1.15 (1.11–1.20)	<0.001	1.26 (1.20–1.33)	<0.001
Social class III	1.58 (2.44–1.72)	<0.001	1.46 (1.38–1.55)	<0.001	1.49 (1.38–1.60)	<0.001
Non-smokers	1		1		1	
Smokers	1.54 (1.49–1.60)	<0.001	1.25 (1.20–1.31)	<0.001	1.20 (1.16–1.25)	<0.001
Yes Mediterranean diet	1		1		1	
Non Mediterranean diet	2.69 (2.33–3.16)	<0.001	3.14 (2.80–3.49)	<0.001	4.92 (4.02–5.83)	<0.001
Yes physical activity	1		1		1	
Non physical activity	6.21 (5.30–7.11)	<0.001	6.95 (5.96–7.95)	<0.001	9.95 (8.60–11.31)	<0.001
Social isolation normal	1		1		1	
Social isolation low	1.98 (1.75–2.22)	<0.001	2.69 (2.45–2.93)	<0.001	3.76 (3.15–4.37)	<0.001

TyG, Triglyceride glucose index. METS-IR, Metabolic score for insulin resistance. SPISE-IR, Single point for insulin sensitivity–insulin resistance. SPISE-IR = 10/SPISE. OR, Odds ratio. CI, Confidence interval.

## Data Availability

All datasets generated and analyzed in the present study are stored securely at ADEMA University School, in accordance with current data protection laws. Data oversight is managed by the institution’s Data Protection Officer, Ángel Arturo López González. Restrictions apply to the datasets. The data underlying this article are not publicly available due to the presence of patient identifiers and the need to protect confidentiality. Data access requests may be directed to the corresponding author.
